# OLDER ADULTS WHO PLAY VIDEO GAMES: WHO ARE THEY AND HOW DO THEY FARE?

**DOI:** 10.1093/geroni/igae098.4246

**Published:** 2024-12-31

**Authors:** Amanda Sonnega, John Sonnega

**Affiliations:** University of Michigan, Ann Arboar, Michigan, United States; Eastern Michigan University, Plymouth, Michigan, United States

## Abstract

Video games for leisure are often considered the purview of younger adults. When older adults are the focus of video game use it is often for rehabilitation or prevention. Growing literature explores the potential of video games to improve cognition and health in older adults but very little that is population-based. Available literature shows that fewer older adults engage in gaming when compared to younger adults but that the older adults play with a similar frequency to younger populations. Internet use provides psychosocial benefits to older adults, and we propose that gaming for leisure does so as well. We use data from the Health and Retirement Study (HRS), a biennial survey of a nationally representative sample of adults over age 50 in the United States begun in 1992. HRS, in an add-on module, asked respondents if they played video games such as X-box or PlayStation (n=1,734). We describe the socio-demographics and other individual differences of respondents who report playing video games. We find that those who report using video games are more likely to be male (64%) and younger than those who do not report using these games (mean age of gamers compared to nongamers is 62 year v. 74 years; p<.01). The gender gap shrinks as adults age. We subsequently examine the relationships between gaming and psychosocial outcomes, and find no difference between gamers and nongamers in depressive symptoms (p=.12). Interestingly, gamers have slightly higher cognitive scores (p<.02) longitudinally. We discuss the potential role of gaming in retirement.

